# Efficacy of Standardized Extract of* Bacopa monnieri* (Bacognize®) on Cognitive Functions of Medical Students: A Six-Week, Randomized Placebo-Controlled Trial

**DOI:** 10.1155/2016/4103423

**Published:** 2016-10-10

**Authors:** Navneet Kumar, L. G. Abichandani, Vijay Thawani, K. J. Gharpure, M. U. R. Naidu, G. Venkat Ramana

**Affiliations:** ^1^Section of Cardiology, Department of Internal Medicine, St John Hospital & Medical Center, Detroit, MI 48236, USA; ^2^Department of Biochemistry, Grant Medical College, Mumbai, India; ^3^People's College of Medical Sciences & Research Centre, Bhanpur, Bhopal 462037, India; ^4^Government Medical College, Nagpur 440 003, India; ^5^Department of Clinical Pharmacology and Therapeutics, Nizam's Institute of Medical Sciences, Hyderabad 500 082, India

## Abstract

*Rationale*.* Bacopa monnieri*, popularly known as Brahmi, has been traditionally used in Ayurveda since ages for its memory enhancing properties. However, data on placebo-controlled trial of* Bacopa monnieri* on intellectual sample is scarce. Hence this study was planned to evaluate the effect of* Bacopa monnieri* on memory of medical students for six weeks.* Objective*. To evaluate the efficacy of* Bacopa monnieri* on memory of medical students with six weeks' administration.* Method and Material*. This was a randomized double blind placebo-controlled noncrossover, parallel trial. Sixty medical students of either gender from second year of medical school, third term, regular batch, were enrolled from Government Medical College, Nagpur, India. Baseline biochemical and memory tests were done. The participants were randomly divided in two groups to receive either 150 mg of standardized extract of* Bacopa monnieri* (Bacognize) or matching placebo twice daily for six weeks. All baseline investigations were repeated at the end of the trial. Students were followed up for 15 days after the intervention.* Results*. Statistically significant improvement was seen in the tests relating to the cognitive functions with use of* Bacopa monnieri*. Blood biochemistry also showed a significant increase in serum calcium levels (still within normal range).

## 1. Introduction

*Bacopa monnieri* (Linn.) Pennell, family Scrophulariaceae [1], is an indigenous plant, found throughout India, Nepal, Sri Lanka, China, Taiwan, Vietnam and Florida, Hawaii, and some other southern states of USA. It is also known as “Brahmi.” Its description in Indian scriptures dates back to 5000 BC. It has been used in Ayurveda since 500 AD as “Medhya Rasayana” [2] for treatment of anxiety, poor memory, epilepsy [3], improvement of cognitive processes, comprehension, memory, and recall [4]. It is currently promoted as a brain tonic and many formulations are available.

Various phytochemical studies have shown its chemical constituents to be alkaloids brahmine, herpestine, nicotine, saponin, monierin, hersaponin [1], Bacosides A1, A2 [5], A3 [6], and B [7], bacogenins A1 to A4 [8], steroids triterpene, and bacosine. Apart from the Bacopasaponins A–F [9, 10], three new triterpenoid glycosides, Bacopasides III–V have been shown by HPLC [11]. Methods for quantitative determination of Bacoside A have been developed by UV spectroscopy [12] and HPLC [13].

*Bacopa* extract has been shown to possess dose dependent free radical scavenging capacity and protective effect on DNA cleavage [14]. Its antioxidant property, which is about half potent to that of vitamin E on weight basis [15], is thought to be responsible for its antistress, immunomodulatory, cognition facilitatory, anti-inflammatory, and antiageing effect [16–23]. Its antilipid peroxidation property is credited to the memory enhancing action and it has been recommended for low dose long-term therapy rather than single high dose administration [15].

Animal studies have shown that it has anxiolytic, cognition enhancing, relaxing, bronchodilatory, antioxidant, anticancer, antidepressant, immunomodulatory, and anti-inflammatory effects [16–24]. Its anxiolytic activity was comparable to benzodiazepine but did not result in any significant motor deficit [16]. It improved acquisition, retention, and retrieval of learned tasks [16–18]. Mouse model shows that its antidepressant properties which may be due to its effect on serotonergic and noradrenergic nervous system [24]. It showed significant memory-promoting effect in animal models of Alzheimer's disease [16]. It has been shown to improve learning and memory in Wister albino rats [25] and even in our other published open label 6-month study in elderly Alzheimer's patients [26].

Trial with a Composite Indian Herbal Preparation (CIHP), which amongst other constituents contained Brahmi, has been shown to be beneficial for soldiers serving at high altitudes and cold areas and in low intensity conflict situations. It improved their physical and mental efficacy [27].

Bacosides A and B improve the transmission of impulses between the neurons. Bacosides regenerate synapses and repair damaged neurons, thereby making it easier to learn and remember new information. Brahmi increases brain serotonin, a neurotransmitter promoting relaxation [5]. Bacosides A and B influence cholinergic system [28]. Bacoside-rich extract reversed the cognitive deficit induced by intracerebroventricularly administered colchicine, a neurotoxin, and that induced by injecting ibotenic acid into the nucleus basalis magnocellularis [29]. The potent antioxidant property exerted by Brahmi has been shown to be due to metal chelation at the initiation level and also as a chain breaker [15]. The mechanism resembles that of EDTA and vitamin E. Standardized extract of* Bacopa monnieri* induced a dose related increase in superoxide dismutase, catalase, and glutathione peroxidase levels in rat prefrontal cortex, striatum, and hippocampus [28]. The antioxidant property, also seen by its protective effect on human fibroblast DNA, suggests its application in diseases where free radicals play a key role [14].

Bacosides A and B were found to be well tolerated in single dose (20–300 mg) as well as multiple doses (100 and 200 mg) for four weeks by healthy human volunteers [30].* Bacopa* significantly improved speed of visual information processing, learning rate, memory consolidation, and decreased anxiety with maximal effects after 12 weeks of administration. The study suggested that it might improve higher order cognitive processes like learning and memory [31].* Bacopa* in syrup form, equivalent to 1 gm dried* Bacopa* daily, for three months in 40 school children aged 6–8 years, showed improvement in immediate memory, perception, and reaction performance without any side effects [32].

Single dose of 300 mg of standardized extract of* Bacopa monniera* failed to show any effects on normal healthy adults [33]. Follow-up study with higher single dose in a double blind, placebo-controlled trial showed improved and sustained cognitive performance [34].

Chronic effects of* Bacopa* when assessed on memory functions in 76 adults from 40 to 60 years' age group, for three months, showed significant effects on retention of new information [35].

In mild cognitive impairment patients, single dose of* Bacopa monnieri* and* Sideritis scardica* extracts showed improvement in d2-concentration test [36].

Most of the reports relate to* Bacopa*'s efficacy on memory and cognition in healthy normal individuals but some also relate to Attention Deficit Hyperactivity Disorder (ADHD) children [37], some of which are ongoing [38].

Unfortunately, only a few randomized, placebo-controlled human trials have been published [39]. A review found only 6 such trials which mainly examined effects of* Bacopa* on memory. Another meta-analysis showed improved cognition and decreased choice reaction time in <10 included trials [40]. There is also paucity of data on effects of* Bacopa* on other cognitive domains like attention, associative abilities, reasoning, transformation, and language comprehension.

Since none has been attempted on a select, exclusive group of participants with high intellectual level, like medical students, we planned this study to see whether the drug is effective in improving cognitive functions in medical students after 6 weeks of treatment.

Our rationale behind this exclusive sample was that medical physicians are known to have a very high intellectual level [41]. So we wanted to see if* Bacopa monnieri* can improve cognition in the group of medical students with already high cognitive functions.

## 2. Method and Material

This was a randomized double blind placebo-controlled, noncrossover, parallel trial conducted on outdoor basis. After Institutional Ethics Committee approval, the students of either gender, second year medical school, third term regular batch from Government Medical College, Nagpur, India were requested to participate in the trial. Detailed information sheet relating to the trial was provided to the students willing to participate. All their questions relating to the trial were answered. They were free to consult peers, doctors, teachers, parents, and so forth, about their participation in the trial. They were told that that their nonparticipation will not affect their student academic life. Students were explained their right to drop out of the trial anytime they wished to, without any questions being asked.

The inclusion criteria were

- being of either gender, in age group 19−22 years,
- having basic computer literacy and exposure to computerized tests,
- volunteering to participate and give signed informed consent.

The exclusion criteria were

- consumption of any memory improving medicines, alcohol, tobacco, or any other Central Nervous System (CNS) acting medicines,
- personal history of suffering from any allergic disease,
- suffering from chronic diseases like hypertension, ischemic heart disease, diabetes, and psychiatric or CNS disorders,
- having abnormal CNS, respiratory, cardiovascular, or per abdominal findings,
- having participated in any medicine trial or donated blood in the past one month,
- using Brahmi in any form, for example, hair oil.

First 60 students found compliant with the inclusion-exclusion criteria were entered in the trial. Informed, written, witnessed consent was obtained from them. A battery of tests was prepared in consultation with clinical psychologists and computer experts. The paper-based tests were developed in collaboration with Psychology Department of Lady Amritabai Daga (LAD) College for Women, Nagpur, India. The computerized tests were administered using NCS Mindomatics software (a psychometric software) prepared by M/s Sristek Consultancy and Nizam's Institute of Mental Sciences, Hyderabad, India. These tests were pretested on five students. The tests were administered to the participants a week before the start of trial for experience. One-week washout was given. On the day of commencement of the trial the same tests were repeated and 5 mL of venous blood was drawn from forearm, in a sterile, deionized glass test tube, for baseline estimation of serum calcium (estimated by method of Baron and Bell), serum total cholesterol (by CHOD-POD method [42]), serum HDL (by enzymatic method [43]), serum triglycerides (by GPO-POD method [44]), and serum LDL (calculated by total cholesterol − HDL − [triglycerides/5]).

The standardized extract of* Bacopa monnieri* (Bacognize) for our trial has been shown to improve some aspects of cognitive functions in a 6-month trial in geriatric Alzheimer's patients [26]. It has also been shown to be safe and have sustained cognitive effects when used for 12 weeks in healthy older adults [45]. Dosage was chosen based on previously reported trials [26, 31, 34, 35].

**Figure 1:**
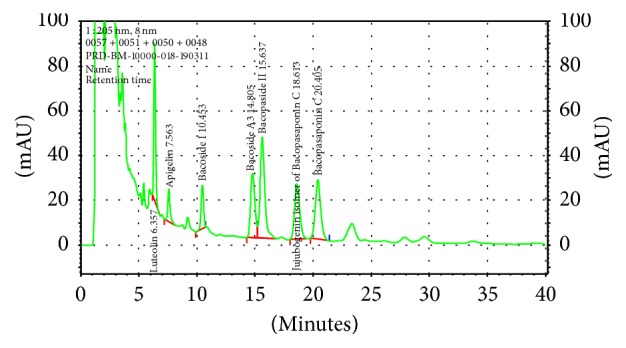
HPLC fingerprint of standardized* Bacopa monnieri* extract (Bacognize) used in the trial.

It was obtained from M/s Pharmanza Herbal Pvt. Ltd., Gujarat, India. Aerial parts of* Bacopa monnieri* were collected from Banks of Howrah River, Kolkata, India. Herb was authenticated by Botanical Survey of India, Jodhpur, India. It was further confirmed by methods given in Indian Pharmacopeia. Plant material was washed with water. After discarding water, the material was dried at 50°C for 12 hours. Dry material was extracted with 4 volumes of methanol twice at 60°C. The methanol extract was concentrated and the material was spray-dried to get powder. The extract contained 45%* Bacopa *saponins when analyzed using UV spectrophotometer [46]. The dosage form was also analyzed using HPLC-PDA (Shimadzu 1100 series). The analytical column (Phenomenex, LUNA C18 (2), 5 *μ*m 150 × 4.6) was used with mobile phase (MP) comprising 0.1% Trifluro acetic acid in acetonitrile, 0.1% Trifluro acetic acid in water (35 : 65 v/v), under isocratic mode of separation. The injection volume was 20 *μ*L, with run time 15 minutes, flow rate 1.5 mL/min, and detector wavelength set at 205 nm. Autosampler carry-over was determined by first injecting the highest calibration standard before a blank sample. Negligible carry-over was observed, as indicated by the lack of* Bacopa* glycosides peaks in the blank sample. A 5 mg/mL concentration sample of standardized* Bacopa monnieri* extract was prepared in methanol and analyzed. Standards for Bacoside A3, Bacoside II, Bacopaside X, and Bacopasaponins C were obtained from Chromadex USA. Total of four bacosides were 11.38% by HPLC (Table 1 and Figure 1).

The participants were randomly given tablet* Bacopa monnieri* 150 mg of standardized extract or matching placebo in identical weight, color, shape, size, and packing to be taken orally two times a day, for 15 days. The participants were impressed to take the medicines regularly and record the miss, if any, and provide the information of missing intake during next visit. The participants were recalled after 15 days for review, ADR monitoring, and refill of medicine. They were instructed to bring back all the unused medicines. Compliance was calculated from returned medicine. 80% consumption was considered to be compliant. All returned medicines were discarded and fresh supply was issued for next 15 days. Such three reviews made up the total duration of therapy 45 days. After completion of the drug run-in, all baseline tests were repeated. The order of posttest was the same as the pretest. The medicine was decoded. The participants were followed up for 15 days after stopping the intervention.

## 3. Neuropsychological Tests

A battery of established auditory and visual neuropsychological tests was chosen aiming to assess immediate recall, recognition, working memory, attention, associative abilities, reasoning, transformation, and language comprehension. These included digit span memory task, paired associate task, logical memory test, memory span for nonsense syllables, finger tapping test, simple and choice reaction time, choice discrimination test, and digit picture substitution test. These tests give results in the form of scores. The higher the score, the better the participants' performance. The details of each test are given below.

### 3.1. Digit Span Memory Task

It involves immediate verbal recall of numbers, varying from 6 to 10 digits, and consists of digit span forwards and backwards. Digit span forwards requires recall of the series of numbers in the same order as given. The test measures are closely related to the efficiency of attention (freedom from distractibility). Digit span backwards requires immediate recall of the spoken numbers in the reverse order. Measuring the elements of working memory, it requires both recall and subsequent manipulation of the incoming information [47]. Digit span is a subset of the WAIS-R, which has exhibited good test-retest reliability, with coefficients 0.70 to 0.89 according to the age group [48]. The two forms employed in our study were derived from the WAIS-R [48], WISC-R [49], WAIS-III [50], and WMS-R [51].

### 3.2. Paired Associate Task

It consists of 10 predetermined word pairs. Participants are first presented with the pairs before the start of the test. In this they are told one member of the pair and are required to verbally give the other part of the pair. Associative abilities enable the participants to connect the stimuli and events. This test requires reasoning and transformation [52–54].

### 3.3. Logical Memory Test (Story Recall)

This test consists of a memory passage. Parallel forms are used in predrug and postdrug testing. The test measures immediate recall of the logical material and language comprehension. High comparability has been reported between the alternate forms [48].

### 3.4. Memory Span for Nonsense Syllables

The test measures immediate memory involving meaningless material with the help of recognition [55]. Recognition can help to measure subtle differences in memory abilities much better than memory recall. Although one may be unable to recall the associated details contained in the previously stated statement, one may still be able to recognize the nonsense syllables.

## 4. Computerized Tests

The following tests were conducted making participants seated in a silent room in front of a computer monitor with NCS Mindomatics software running along with a specially designed response keyboard.

### 4.1. Finger Tapping Test

This test provides the information about motor performance. With the duration of the test being 8 sec, the participant has to continuously tap on the enter button on the response keyboard in quick succession. The results are presented as average reaction time in milliseconds and total number of successful clicks.

### 4.2. Simple Reaction Test

This is performed to assess attention and sensory-motor performance of brain. On press of the start button the test time begins and a picture of a boy appears randomly in the center of a screen, for 20 times. On visualizing the boy picture the individual has to press the “BOY” symbol button on response keyboard as quickly as possible. The picture stays on the screen for one second and the time gap between subsequent appearances of picture is 1.5–2.5 seconds. Results are given as average reaction time.

### 4.3. Choice Reaction Test

This is used to assess the attention and sensory-motor performance of brain and estimate the psychomotor speed. On the click of start button the test time starts and a picture of a boy or girl appears randomly on the center of the monitor screen. Both the pictures randomly appear 20 times, during the test span. Participant has to press BOY symbol button on response keyboard as quickly as possible only when the boy picture appears. Every time the picture remains on the screen for one second and the time gap between subsequent appearances is between 1.5–2.5 sec. Results are presented in terms of average reaction time, correct hits, and wrong attempts.

### 4.4. Choice Discrimination Test

This is used to assess the attention and sensory-motor performance of brain and estimate the psychomotor speed. On the click of start button the test time begins and a picture of a boy or girl appears randomly in the center of monitor. Ten times Boy picture and ten times Girl picture appear in random order during the test period. Matching the monitor sighting, the participant has to correctly press the BOY symbol button or GIRL symbol button on response keyboard as quickly as possible. Each time the picture remains on the screen for one second and the time gap between subsequent appearances of picture is between 1.5 and 2.5 sec.

### 4.5. Digit Picture Substitution Test (Symbol Digit Modalities Test)

It requires substitution of numbers for respective target pictures. In this test the upper panel of the screen shows numbers 1 to 9 with corresponding target picture. Participant has to carefully concentrate and remember each digit and its respective picture. On click of the start button the pictures shown in the panel start appearing randomly in the center of the screen. Participant has to press the corresponding digit as quickly as possible on the response keyboard thus substituting as many pictures as possible in 90 sec. This test calculates the reaction time, total number of attempts, correct attempts, and wrong attempts. Manual speed and agility contribute significantly to performance [56].

Students' “*t*”-test was used to determine statistical significance.

## 5. Results

Two participants were dropped due to noncompliance. Some participants did not show up for the posttest while some blood samples could not be processed due to hemolysis. Hence the final sample in the two groups is not the same.

When the demographic characteristics of the participants were analyzed, it was seen that there was no difference between the two intervention groups.

Table 2 shows that there was significant increase in the serum calcium (*p* < 0.05) with 6-week administration of* Bacopa monnieri* and the increased levels were within the normal range. Serum total cholesterol levels showed significant decrease in the* Bacopa* group (*p* < 0.05) but on comparison with the placebo arm, it was statistically nonsignificant. Serum HDL levels showed significant increase (*p* < 0.05) in the* Bacopa* group but when this was compared with placebo group it was nonsignificant. There was no statistical difference between other blood parameters studied by us.

From the various neuropsychological tests conducted (Table 3) digit span backwards and logical memory test showed statistically significant improvement in* Bacopa* group when compared with the placebo treated group.

## 6. Discussion

Efficacy of* Bacopa monnieri* extract on memory of intellectual sample of normal healthy medical students was evaluated after six weeks' oral administration in the dose of 150 mg twice daily. The results show a significant increase in the serum calcium levels in* Bacopa* group and this increase was also significant when compared with the placebo group. The raised calcium levels remained within the normal range (9–11 mg/dL). However, calcium antagonistic activity of* Bacopa* on the vascular and intestinal smooth muscles of rabbit and guinea pig has been reported by others [57].

The lipid profile showed that with* Bacopa* the serum cholesterol decreased and serum HDL levels increased but these changes were not statistically significant when compared with the control group. We admit that the confounding factors like diet, stress, and anxiety were not eliminated since the students continued with their routine college lifestyle.

The neuropsychological measures for efficiency of attention, freedom from distractibility, and working memory improved significantly with use of* Bacopa* (*p* < 0.05) as was seen by digit span backwards test. The logical memory test, known to be a measure of immediate recall of logical material and language comprehension, also significantly improved (*p* < 0.05) with* Bacopa monnieri*. These tests required the participants to learn new material and recall it a short time later. The digit span test in fact showed mixed response. The forward component showed significant improvement in the active group but it was nonsignificant on comparison with placebo group. This indicates that* Bacopa* does reduce the distractibility of participants to some extent. The backwards component, which turned out to be significantly revealed that the improvement in the working memory was significant. This indicates that Brahmi enhances both the immediate recall and its subsequent manipulation as indicated by the participants' ability to reproduce a given number in reverse order. The rest of the neuropsychological tests did not show any significant improvement after* Bacopa* administration.

## 7. Conclusion

Our study further adds to the increasing scientific evidence supporting cognitive enhancement effects of* Bacopa monnieri* in humans. However, our study is unique in the aspect that it was done in a randomized, placebo-controlled trial on a group of individuals with already high cognitive abilities. Also* Bacopa monnieri* extract in the dose of 300 mg daily produced significant effect on some components of memory with only 6 weeks of administration. This has been reported previously only with 12 weeks' therapy in other studies on normal individuals [31, 35]. Since our study was only limited to administrating* Bacopa monnieri* for only 6 weeks, we recommend future long-term studies to study long-term effects of* Bacopa monnieri*.

Our study also tested subjects' other cognitive functions apart from memory which may be useful in evaluating* Bacopa monnieri*'s cognitive effects in future studies.

The antioxidant effect, action on calcium channels, and acetylcholine level have been credited for the action of* Bacopa*. We also found significant increase in serum calcium levels after* Bacopa* administration (still within normal range).

Memory and learning, being complex processes, have several components; multiple neurotransmitters and factors are involved. We also recommend further studies to investigate role of calcium in explaining the nootropic effects of* Bacopa monnieri*.

## Figures and Tables

**Table 1:** HPLC analytical data of standardized *Bacopa monnieri* extract (Bacognize) used in the trial.

Analytes	Result	Unit
Bacoside A3	4.57	%w/w
Bacopaside II	2.13	%w/w
Bacopaside X	1.70	%w/w
Bacopasaponin C	2.98	%w/w
*Total Bacopasaponin [identified]*	*11.38*	*%w/w*
*Total Bacopasaponin [identified + unidentified]*	*15.57*	*%w/w*

**Table 2:** Effect of *Bacopa monnieri* (300 mg/day) on blood biochemistry (mean values ± SD).

Biochemical investigations	*Bacopa* group		Placebo group		“*p*” value
	(*n* = 26)		(*n* = 18)		
	Before drug	After drug	Before drug	After drug	
Serum calcium (mg/dL)	9.9 ± 0.5	10.2 ± 0.6	10.2 ± 0.8	9.9 ± 0.5	**<**0.05^*∗*^
Serum Triglycerides (mg%)	128.6 ± 40.3	130.4 ± 42.3	144.6 ± 39.4	134.6 ± 37	>0.05
Serum cholesterol (mg%)	142.8 ± 24.8	135.8 ± 23.6	146.7 ± 26.2	134.7 ± 17.9	>0.05
Serum HDL (mg%)	39.6 ± 8.4	45.1 ± 10.4	40.5 ± 9.4	41 ± 8.7	>0.05
Serum LDL (mg%)	71.2 ± 19.0	71.7 ± 18.6	77.3 ± 16.5	66.2 ± 12.0	>0.05

^*∗*^On comparing *Bacopa* group with placebo group by using unpaired Student's “*t*”-test.

**Table 3:** Effect of *Bacopa monnieri* (300 mg/day) on neuropsychological tests (mean scores ± SD).

Neuropsychological task	*Bacopa* group		Placebo group		“*p*” value
	(*n* = 28)		(*n* = 14)		
	Before drug	After drug	Before drug	After drug	
Digit span forwards	7.23 ± 1.11	7.85 ± 0.83	7.83 ± 1.19	7.73 ± 0.23	>0.05
Digit span backwards	8.1 ± 1.3	8.3 ± 0.9	9.1 ± 1.0	8.3 ± 0.8	**<**0.05^*∗*^
Paired associate task	8 ± 1.3	7.7 ± 1.4	8.1 ± 1.1	7.9 ± 1.3	>0.05
Logical memory test	3.4 ± 2.6	5.2 ± 4.5	5.5 ± 2.6	4.1 ± 3.8	**<**0.05^*∗*^
Memory span for nonsense syllables	5.4 ± 3.3	6.9 ± 2.4	4.7 ± 2.3	6.1 ± 2.9	>0.05
Finger tapping test	152.28 ± 15.75	154.56 ± 13.71	157.60 ± 14.89	158.48 ± 27.89	>0.05
Simple reaction time	221.88 ± 53.17	221.01 ± 52.16	213.13 ± 33.6	212.49 ± 33.62	>0.05
Choice reaction time	350.07 ± 52.08	340.03 ± 62.26	342.39 ± 67.54	340.95 ± 64.45	>0.05
Choice discrimination test	383.70 ± 52.54	387.06 ± 53.47	374.02 ± 51.46	379.44 ± 58.32	>0.05
Digit picture substitution test	1492.69 ± 368.58	1467.78 ± 556.55	1465.94 ± 293.94	1325.44 ± 207.07	>0.05

^*∗*^On comparing *Bacopa* group with placebo group by using unpaired Student's “*t*”-test.

## Data Access

Raw data of this trial or the results have not been made available to the pharmaceutical company which supplied the medicines.

## References

[1] Sharma P. C., Yelne M. B., Dennis T. J. (2000). Volume I. *Database on Medicinal Plants Used in Ayurveda*.

[2] Sharma P. V. (1998). *Dravyaguna-Vijñāna*.

[3] Badmaev V. (1998). *Bacopin (Bacopa monnieri): A Memory Enhancer from Ayurveda*.

[4] Mukherjee G. D., Dey C. D. (1966). Clinical trial on Brahmi. I. *Journal of Experimental Medical Sciences*.

[5] Rastogi S., Kulshreshtha D. K. (1998). Bacoside A2—A triterpenoid saponin from *Bacopa monniera*. *Indian Journal of Chemistry B*.

[6] Rastogi S., Pal R., Kulshreshtha D. K. (1994). Bacoside A3—A triterpenoid saponin from Bacopa monniera. *Phytochemistry*.

[7] Basu N., Rastogi R. P., Dhar M. L. (1967). Chemical examination of Bacopa monniera Wettst: part III, bacoside B. *Indian Journal of Chemistry*.

[8] Chandel R. S., Kulshreshtha D. K., Rastogi R. P. (1977). Bacogenin-A3: a new sapogenin from *Bacopa monniera*. *Phytochemistry*.

[9] Garai S., Mahato S. B., Ohtani K., Yamasaki K. (1996). Dammarane-type triterpenoid saponins from *Bacopa monniera*. *Phytochemistry*.

[10] Mahato S. B., Garai S., Chakravarty A. K. (2000). Bacopasaponins E and F: Two jujubogenin bisdesmosides from Bacopa monniera. *Phytochemistry*.

[11] Chakravarty A. K., Garai S., Masuda K., Nakane T., Kawahara N. (2003). Bacopasides III-V: three new triterpenoid glycosides from Bacopa monniera. *Chemical and Pharmaceutical Bulletin*.

[12] Pal R., Sarin J. P. S. (1992). Quantitative determination of bacosides by UV-spectrophotometry. *Indian Journal of Pharmaceutical Sciences*.

[13] Pal R., Dwivedi A. K., Singh A., Kulshreshtha D. K. (1996). Quantitative determination of bacosides by HPLC. *Indian Journal of Pharmaceutical Sciences*.

[14] Russo A., Izzo A. A., Borrelli F., Renis M., Vanella A. (2003). Free radical scavenging capacity and protective effect of *Bacopa monniera* L. on DNA damage. *Phytotherapy Research*.

[15] Tripathi Y. B., Chaurasia S., Tripathi E., Upadhyay A., Dubey G. P. (1996). Bacopa monniera Linn. as an antioxidant: mechanism of action. *Indian Journal of Experimental Biology*.

[16] Bhattacharya S. K., Ghosal S. (1998). Anxiolytic activity of a standardized extract of Bacopa monniera: An Experimental Study. *Phytomedicine*.

[17] Singh H. K., Dhawan B. N. (1982). Effect of *Bacopa monniera* Linn. (Brāhmi) extract on avoidance responses in rat. *Journal of Ethnopharmacology*.

[18] Singh H. K., Rastogi R. P., Srimal R. C., Dhawan B. N. (1988). Effect of bacosides A and B on avoidance responses in rats. *Phytotherapy Research*.

[19] Dar A., Channa S. (1997). Relaxant effect of ethanol extract of *Bacopa monniera* on trachea, pulmonary artery and aorta from rabbit and guinea-pig. *Phytotherapy Research*.

[20] Dar A., Channa S. (1997). Relaxant effect of ethanol extract of *Bacopa monniera* on trachea, pulmonary artery and aorta from rabbit and guinea-pig. *Phytotherapy Research*.

[21] Elangovan V., Govindasamy S., Ramamoorthy N., Balasubramanian K. (1995). In vitro studies on the anticancer activity of Bacopa monnieri. *Fitoterapia*.

[22] Dahanukar S., Thatte U. (1997). Current status of ayurveda in phytomedicine. *Phytomedicine*.

[23] Jain P., Khanna N. K., Trehan N., Pendse V. K., Godhwani J. L. (1994). Antiinflammatory effects of an Ayurvedic preparation, Brahmi Rasayan, in rodents. *Indian Journal of Experimental Biology*.

[24] Girish C., Oommen S., Vishnu R. (2016). Evidence for the involvement of the monoaminergic system in the antidepressant-like activity of methanolic extract of Bacopa monnieri in albino mice. *International Journal of Basic and Clinical Pharmacology*.

[25] Ramadas D., Ravishankar M., Shwetha S., Srinivas L. (2016). The learning and memory enhancing properties of *Bacopa monnieri* plant leaves protein: a systematic study in Wister Albino rats model system. *Scholars Academic Journal of Biosciences*.

[26] Goswami S., Saoji A., Kumar N., Thawani V., Tiwari M., Thawani M. (2011). Effect of Bacopa monnieri on cognitive functions in Alzheimer's disease patients. *International Journal of Collaborative Research on Internal Medicine and Public Health*.

[27] Lalith Singh T. (2004). *A Herbal Stress Buster for Soldiers*.

[28] Bhattacharya S. K., Bhattacharya A., Kumar A., Ghosal S. (2000). Antioxidant activity of *Bacopa monniera* in rat frontal cortex, striatum and hippocampus. *Phytotherapy Research*.

[29] Bhattacharya S. K., Kumar A., Ghosal S., Siva Sanka D. V. (2001). Effect of Bacopa monniera on animal models of Alzheimer’s disease and perturbed central cholinergic markers of cognition in rats. *Molecular Aspects of Asian Medicines*.

[30] Asthana O. P., Srivastava J. S., Ghatak A., Gaur S. P. S., Dhawan B. N. (1996). Safety and tolerability of Bacosides A and B in healthy human volunteers. *Abstracts of Annual Conference of Indian Journal of Pharmacology*.

[31] Stough C., Lloyd J., Clarke J. (2001). The chronic effects of an extract of *Bacopa monniera* (Brahmi) on cognitive function in healthy human subjects. *Psychopharmacology*.

[32] Sharma R., Chaturvedi C., Tewari P. V. (1987). Efficacy of bacopa monnieri in revitalizing intellectual functions in children. *Journal of Research and Education in Indian Medicine*.

[33] Nathan P. J., Clarke J., Lloyd J., Hutchison C. W., Downey L., Stough C. (2001). The acute effects of an extract of *Bacopa monniera* (Brahmi) on cognitive function in healthy normal subjects. *Human Psychopharmacology: Clinical and Experimental*.

[34] Downey L. A., Kean J., Nemeh F. (2013). An acute, double-blind, placebo-controlled crossover study of 320 mg and 640 mg doses of a special extract of *Bacopa monnieri* (CDRI 08) on sustained cognitive performance. *Phytotherapy Research*.

[35] Roodenrys S., Booth D., Bulzomi S., Phipps A., Micallef C., Smoker J. (2002). Chronic effects of Brahmi (*Bacopa monnieri*) on human memory. *Neuropsychopharmacology*.

[36] Dimpfel W., Schombert L., Biller A. (2016). Psychophysiological effects of *Sideritis* and *Bacopa* extract and three combinations thereof—a quantitative EEG study in subjects suffering from mild cognitive impairment (MCI). *Advances in Alzheimer's Disease*.

[37] Srivastava J. S., Asthana O. P., Gupta R. C. (2002). Double blind placebo controlled randomized study of standardized bacopa monniera extract in children with attention deficit hyperactivity disorder. *Indian Journal of Psychiatry*.

[38] Kean J. D., Kaufman J., Lomas J. (2015). A randomized controlled trial investigating the effects of a special extract of *Bacopa monnieri* (CDRI 08) on hyperactivity and inattention in male children and adolescents: BACHI Study Protocol (ANZCTRN12612000827831). *Nutrients*.

[39] Pase M. P., Kean J., Sarris J., Neale C., Scholey A. B., Stough C. (2012). The cognitive-enhancing effects of bacopa monnieri: a systematic review of randomized, controlled human clinical trials. *Journal of Alternative and Complementary Medicine*.

[40] Kongkeaw C., Dilokthornsakul P., Thanarangsarit P., Limpeanchob N., Scholfield C. N. (2014). Meta-analysis of randomized controlled trials on cognitive effects of *Bacopa monnieri* extract. *Journal of Ethnopharmacology*.

[41] Hauser R. M. (2002). *Meritocracy, Cognitive Ability, and the Sources of Occupational Success*.

[42] Allain C. C., Poon L. S., Chan C. S. G. (1974). Enzymatic determination of total serum cholesterol. *Clinical Chemistry*.

[43] Gorden T., Castelli W. P., Hjorltand M. C. (1977). Enzymatic determination of HDL Cholesterol. *American Journal of Medicine*.

[44] Foosati P., Prencipe L. (1982). Serum triglycerides determined colorimetrically with an enzyme that produces hydrogen peroxide. *Clinical Chemistry*.

[45] Hingorani L., Patel S., Ebersole B. (2012). Sustained cognitive effects and safety of HPLC-standardized *Bacopa monnieri* extract: a randomized, placebo controlled clinical trial. *Planta Medica*.

[46] Rajpal V. (2002). *Standardization of Botanicals Botanicals (Testing and Extraction Methods of Medicinal Herbs)*.

[47] Lezak M. D. (1995). *Neuropsychological Assessment*.

[48] Wechsler D. (1981). *WAIS-R Manual*.

[49] Wechsler D. (1974). *Wechsler Memory Scale Manual*.

[50] Wechsler D. (1991). *Wechsler Intelligence Scale for Children*.

[51] Wechsler D. (1987). *Wechsler Memory Scale Revised Manual*.

[52] Jensen A. (1969). How much can we boost IQ and scholastic achievement. *Harvard Educational Review*.

[53] Jensen A. R., Hellmuth J. (1970). Can we and should we study race differences?. *Disadvantaged Child*.

[54] Jensen A. R. (1987). Psychometric g as a focus of concerted research effort. *Intelligence*.

[55] Ebbinghaus H. (1985). *Memory: A Contribution to Experimental Psychology*.

[56] Schear J. M., Sato S. D. (1989). Effects of visual acuity and visual motor speed and dexterity on cognitive test performance. *Archives of Clinical Neuropsychology*.

[57] Dar A., Channa S. (1999). Calcium antagonistic activity of Bacopa monniera on vascular and intestinal smooth muscles of rabbit and guinea-pig. *Journal of Ethnopharmacology*.

